# Household Transmission of Group A Streptococcus Necrotizing Fasciitis

**DOI:** 10.5435/JAAOSGlobal-D-21-00171

**Published:** 2022-08-03

**Authors:** Michael Markowitz, Stephanie Kwan, Jonas L. Matzon

**Affiliations:** From the Department of Orthopaedic Surgery (Dr. Markowitz, Dr. Kwan), Rowan University SOM, Stratford, NJ (Dr. Markowitz, Dr. Kwan), and Orthopaedic Surgery (Dr. Matzon), Rothman Orthopaedic Institute—Hand, Wrist, Elbow, & Microvascular Surgery, Sidney Kimmel Medical College at Thomas Jefferson University, Rothman Institute, Philadelphia, PA.

## Abstract

A healthy 40-year-old woman was diagnosed with necrotizing fasciitis 2 days after her husband's death from the same infectious process. Prompt identification and immediate surgical intervention prevented a similar result in this patient. Additional investigation into both patients' medical records found the inciting organism to be group A streptococcus. Although the exact mechanism of inoculation is unknown, the spread of this infection within a household prompts the question of whether antibiotic prophylaxis should be given among close contacts in future cases of necrotizing fasciitis.

Necrotizing fasciitis (NF) is a life-threatening soft-tissue infection that rapidly spreads through myofascial planes and surrounding soft tissues.^1,2^ These infections can occur after both major trauma and minor skin breaches, with the most common presenting symptoms being swelling, pain, and erythema.^3^ NF is classified by causative organisms, where type I is polymicrobial, type II is monomicrobial (usually group A streptococcus [GAS]), and type III is through marine vibrios.^2-4^ Specifically, GAS is the causative organism for approximately 10% of NF cases annually.^4^ Almost half of these cases lack an identifiable portal of entry but may be related to an antecedent hematoma or muscle strain.^5,6^

In the United States, there are only 700 to 1200 cases/year of type II NF according to the Centers for Disease Control.^7^ Group A streptococcus is highly virulent, and those exposed to an infected individual are up to 50 to 2000 times greater risk of developing infectious manifestations.^8,9^ Two prospective studies identified 5 cases of household transmission of GAS non-NF infections resulting in NF over a 4-year period; however, no recommendations for chemoprophylaxis were made.^10^ We present two healthy family members who contracted GAS NF within a 1-week period. Our aim is to evaluate the transmissibility of GAS NF and the potential need for chemoprophylaxis of close contacts. The patient consented for this case to be published.

## Case Report

A 40-year-old healthy man presented to an outside hospital with acute right shoulder pain and “bruising” from moving furniture the day prior. He was discharged home with a suspected rotator cuff injury but continued to deteriorate. Three days later, he returned to the outside hospital with diffuse erythema and crepitus about the arm and chest wall. He was diagnosed with septic shock secondary to GAS NF and subsequently died. According to the family and medical record review, the patient had no identifiable risk factors or medical history predisposing him to NF. No recommendations were made by the outside hospital regarding chemoprophylaxis for the patient's family.

**Figure 1:**
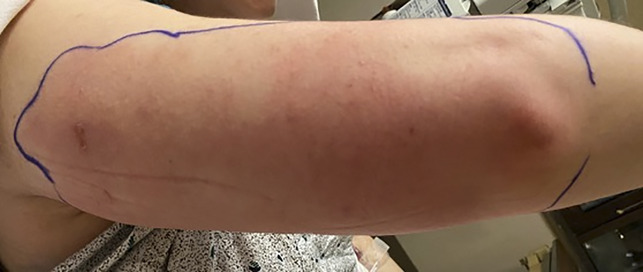
Clinical photograph demonstrating initial presentation with demarcated area of erythema over the posterior aspect of the right upper extremity.

Two days after his death, his wife presented to our hospital with right upper extremity cellulitis extending from the posterior aspect of her shoulder to her elbow (Figure 1). She was a similarly healthy, 40-year-old woman who reported that her symptoms began as a small pustule. She noted progressive erythema and edema that rapidly worsened. Concomitantly, she became increasingly lethargic and eventually febrile. She denied any recent trauma to the skin, insect bites, or history of intravenous drug use. The patient worked a desk job and reported no occupational exposures. Her medical history was only notable for anxiety for which she took alprazolam and smoked marijuana.

On initial evaluation, the patient was found to be hypotensive, tachycardic, diaphoretic, and febrile (Tmax: 102.4°F). Initial laboratory studies demonstrated substantial metabolic derangements with elevated white blood cell count, C-reactive protein, and lactic acid (Table 1). She underwent investigation for an underlying causative etiology using chest radiograph, urinalysis/urine drug screen, COVID-19 polymerase chain reaction (PCR), CT abdomen/pelvis, and blood cultures. All of these tests resulted negative. Rapidly, the patient required vasopressor therapy for maintenance of hemodynamic stability. She was admitted to the ICU in septic shock with the only presumed source being the cellulitis. Orthopaedic surgery and general surgery were consulted.

**Table 1:** Laboratory Risk Indicator for the Necrotizing Fasciitis (LRINEC) Score

Variable (Unit)	Score
CRP (mg/dL)	
<15	0
≥15	4
WBC (B/L)	
<15	0
15-25	1
>25	2
Hemoglobin (g/dL)	
>13.5	0
11-13.5	1
<11	2
Na (mmol/L)	
≥135	0
<135	2
Creatinine (mg/dL)	
≤1.6	0
>1.6	2
Glucose (mg/dL)	
≤108	0
>180	1

CRP = C-reactive protein, WBC = white blood cell.

A score ≥6 has a positive predictive value of 92.0% and a negative predictive value of 96.0%.

Radiographs and CT imaging of the right upper extremity demonstrated subcutaneous edema without signs of gas in the tissues or focal fluid collection. Using the laboratory risk indicator for the NF score (Table 1), the patient scored 10 (Table 2), giving her a positive predictive value of greater than 92% for NF.^11^ Ultimately, due to the patient's rapid clinical decline, orthopaedic surgery took the patient emergently to the operating room for irrigation and débridement.

**Table 2:** Laboratory Data From Initial Patient Presentation

Variable	Value	Normal
CRP	31.5	≤0.50 mg/dL
WBC	16.0	3.7-10.5 B/L
Hemoglobin	11.3	11.7-15.0 g/dL
Na	130	133-145 mmol/L
Creatinine	2.69	0.40-1.10 mg/dL
Glucose	108	70-105 mg/dL

CRP = C-reactive protein, WBC = white blood cell.

Values used to calculate a LRINEC score of 10.

**Figure 2:**
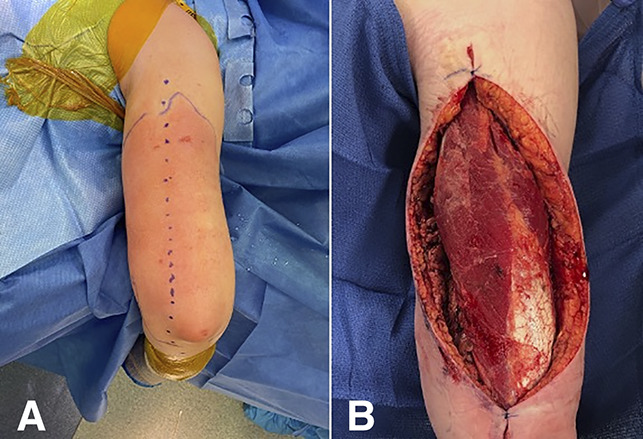
**A**, Clinical photograph demonstrating progression of cellulitis preoperatively with planned surgical incision marked. **B**, Clinical photograph status post extensive irrigation and débridement. The wound was left open with placement of wound vacuum therapy.

**Figure 3:**
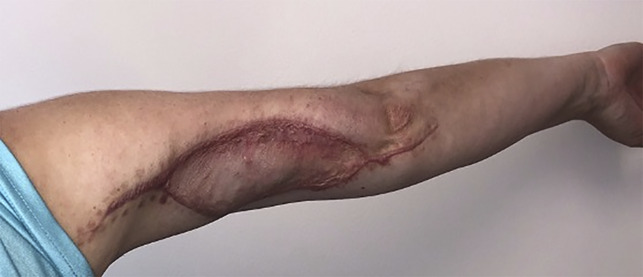
Clinical photograph at the postoperative follow-up demonstrating healing split thickness skin grafting.

In the operating room, a 30-cm incision was made on the posterior aspect of the arm, which immediately revealed “dishwater”-appearing fluid, liquefaction of adipose tissue, and necrosis of the surrounding subcutaneous soft tissues and fascia (Figure 2, A). Tissue and fluid cultures were obtained, which grew GAS. After radical débridement and copious irrigation, the wound was not amenable to closure and required wound vacuum therapy (Figure 2, B). The patient was maintained on broad-spectrum antibiotics consisting of vancomycin, piperacillin-tazobactam, clindamycin, and adjunctive intravenous immunoglobulin (IVIG). She returned for two additional surgical débridements to ensure adequate eradication of the infection. Nine days after initial presentation, the patient underwent split-thickness skin graft from the thigh to the arm (Figure 3). She was discharged from the hospital in stable condition the next day.

Discussion with infectious disease specialists at our facility led to the recommendation of chemoprophylaxis for all members of the household with a 10-day course of clindamycin and sanitation of all shared surfaces in the home. No other family member contracted GAS NF.

## Discussion

NF is a severe infection that requires emergent surgical intervention to prevent substantial morbidity and mortality.^3,4,5,6,10,12^ Nonspecific symptoms of type II NF, caused by GAS, range from localized pain to gastrointestinal symptoms, and 31% of patients are given an initial diagnosis of cellulitis.^4-6,12,13^ Most of these cases lack an identifiable inciting event but quickly progress to a life-threatening infection.^13,14^

Group A streptococcus is highly virulent, and exposed individuals have up to 2000 times of greater risk of becoming symptomatic.^8,9^ Since NF can present with no obvious inciting injury or trauma, it can be mistaken for a more benign disease process, as seen with the husband's presentation. Given its ability to spread to close contacts, families with recent exposure must be properly educated about the signs and symptoms. In addition, patients should seek treatment urgently if they require increasing analgesic with new onset cellulitis to avoid delays in care.^2,13^ Although the mode of transmission is poorly understood, routine chemoprophylaxis for close contacts is not currently recommended unless they have substantial risk factors, such as age older than 65 years, heart disease, diabetes, and/or cancer.^9,14^ Our patient was not given chemoprophylaxis after the recent death of her husband because the causative organism in her husband's death was not identified until after her surgical intervention. Moreover, she did not have any risk factors.

The use of chemoprophylaxis for close contacts of patients with GAS infections is controversial and varies between countries. The Centers for Disease Control has recommended against routine antibiotic prophylaxis, preferring instead a strategy of maintaining a heightened index of suspicion in the 30 days after a severe GAS infection.^9,15^ Concern for the development of drug resistant organisms is often a factor cited in limiting antibiotic usage, but this should be weighed against the potential to decrease morbidity by prevention of contracting NF or other GAS infections.^3,9,10,11,12^ Aside from NF, GAS is the causative organism for other conditions including strep throat, scarlet fever, impetigo, cellulitis, and rheumatic fever. One case study evaluating healthcare workers (HCWs) exposed to a patient with GAS NF demonstrated that nearly 30% of them were infected with GAS and experienced fever, sore throat, and malaise. All of the identified symptomatic HCWs were subsequently treated, and none developed NF.^16^ It is unknown whether these infections could have been prevented with chemoprophylaxis. de Aleimda Torres et al^8^ cultured close contacts of a patient that died from GAS NF, treated all individuals that had positive cultures with oral amoxicillin, and reported no subsequent cases of GAS NF.

In our case, the patient was exposed to her husband with GAS NF, did not receive any chemoprophylaxis, and subsequently developed NF through either direct inoculation or contact with shared surfaces. As far as we are aware, the household transmission of GAS resulting in two cases of NF in healthy individuals has only been reported once in the infectious disease literature.^17^ Unlike our case, both patients had open wounds which could have predisposed to transmission. Moreover, it is imperative that the surgical specialties (Orthopaedic Surgery/General Surgery/Urology) who initially manage these patients are aware of this risk. Knowledge of the devastating nature of NF and the virulence of GAS in particular will allow for appropriately timed chemoprophylaxis. Moving forward, additional investigation is necessary to identify risk factors that are predictive for transmission. This would help to determine which close contacts of patients are the most appropriate candidates for chemoprophylaxis while minimizing the risk of the undesirable effects from antibiotic therapy.

## Conclusion

NF secondary to GAS is a severe infectious process that requires emergent surgical intervention. Although it may present as an indolent, benign cellulitis, high suspicion must remain if there are worsening symptoms or hemodynamic instability in these patients. GAS can be transmitted across close contacts and manifest as NF, so chemoprophylaxis consisting of penicillin or clindamycin should be considered for close contacts.^17^
